# Mechanism of Eravacycline Resistance in Clinical *Enterococcus faecalis* Isolates From China

**DOI:** 10.3389/fmicb.2020.00916

**Published:** 2020-05-25

**Authors:** Zewen Wen, Yongpeng Shang, Guangjian Xu, Zhangya Pu, Zhiwei Lin, Bing Bai, Zhong Chen, Jinxin Zheng, Qiwen Deng, Zhijian Yu

**Affiliations:** ^1^Shenzhen Key Laboratory for Endogenous Infections, Department of Infectious Diseases, Shenzhen Nanshan People’s Hospital, The 6th Affiliated Hospital of Shenzhen University Health Science Center, Shenzhen, China; ^2^Quality Control Center of Hospital Infection Management of Shenzhen, Shenzhen Nanshan People’s Hospital, Guangdong Medical University, Shenzhen, China; ^3^Key Laboratory of Viral Hepatitis of Hunan Province, Department of Infectious Diseases, Xiangya Hospital, Central South University, Changsha, China

**Keywords:** Eravacycline, *Enterococcus faecalis*, heteroresistance, tetracycline resistance genes, resistance mechanism

## Abstract

Opportunistic infections caused by multidrug-resistant *Enterococcus faecalis* strains are a significant clinical challenge. Eravacycline (Erava) is a synthetic fluorocycline structurally similar to tigecycline (Tige) that exhibits robust antimicrobial activity against Gram-positive bacteria. This study investigated the *in vitro* antimicrobial activity and heteroresistance risk of Eravacycline (Erava) in clinical *E. faecalis* isolates from China along with the mechanism of Erava resistance. A total of 276 non-duplicate *E. faecalis* isolates were retrospectively collected from a tertiary care hospital in China. Heteroresistance to Erava and the influence of tetracycline (Tet) resistance genes on Erava susceptibility were examined. To clarify the molecular basis for Erava resistance, *E. faecalis* variants exhibiting Erava-induced resistance were selected under Erava pressure. The relative transcript levels of six candidate genes linked to Erava susceptibility were determined by quantitative reverse-transcription PCR, and their role in Erava resistance and heteroresistance was evaluated by *in vitro* overexpression experiments. We found that Erava minimum inhibitory concentrations (MICs) against clinical *E. faecalis* isolates ranged from ≤0.015 to 0.25 mg/l even in strains harboring Tet resistance genes. The detection frequency of Erava heteroresistance in isolates with MICs ≤ 0.06, 0.125, and 0.25 mg/l were 0.43% (1/231), 7.5% (3/40), and 0 (0/5), respectively. No mutations were detected in the 30S ribosomal subunit gene in Erava heteroresistance-derived clones, although mutations in this subunit conferred cross resistance to Tige in Erava-induced resistant *E. faecalis*. Overexpressing RS00630 (encoding a bone morphogenetic protein family ATP-binding cassette transporter substrate-binding protein) in *E. faecalis* increased the frequency of Erava and Tige heteroresistance, whereas RS12140, RS06145, and RS06880 overexpression conferred heteroresistance to Tige only. These results indicate that Erava has potent *in vitro* antimicrobial activity against clinical *E. faecalis* isolates from China and that Erava heteroresistance can be induced by RS00630 overexpression.

## Introduction

*Enterococcus faecalis* is a Gram-positive facultative anaerobe that normally exists as a commensal microbe colonizing the gastrointestinal or urinary tract of humans and animals (Arias and Murray, 2012). *E. faecalis* is an important nosocomial pathogen that is linked to various infections, including urinary tract infection, sepsis, endocarditis, and peritonitis (Arias and Murray, 2012; Mercuro et al., 2018). *E. faecalis* exhibits both intrinsic and acquired resistance to several commonly used antibiotics, such as aminoglycosides and macrolides, and can serve as a model for the emergence of antibiotic resistance (Arias and Murray, 2012). Given the increasing number of reports of multidrug resistant *E. faecalis* strains, including those resistant to vancomycin (VAN) and linezolid (LZD), there is an urgent need to develop effective measures to treat infections caused by *E. faecalis* (Van Harten et al., 2017; Sparo et al., 2018; Fiore et al., 2019; García-Solache and Rice, 2019).

Eravacycline (Erava) is a novel fluorocycline antibacterial agent belonging to the tetracycline (Tet) drug class (Grossman et al., 2012). Erava has robust antimicrobial activity against a broad range of microorganisms, including Gram-positive, Gram-negative, anaerobic, and atypical bacteria (Sutcliffe et al., 2013; Livermore et al., 2016; Monogue et al., 2016; Zhanel et al., 2016). Intravenous Erava is newly approved in several countries for the treatment of adult patients with complicated intra-abdominal infections (cIAIs) caused by enteric microorganisms, including multidrug resistant, difficult-to-treat *enterococci* infection (Lee and Burton, 2019; Scott, 2019; Alosaimy et al., 2020). Against *E. faecalis* clinical isolates collected globally (Hackel et al., 2013; Sutcliffe et al., 2013; in 2013–2015; Olesky et al., 2017), in Canada (CANWARD surveillance study; 2014–2015; Zhanel et al., 2018), and the USA (2013–2016; Bouchillon et al., 2018), Erava exhibited potent *in vitro* activity, including against vancomycin-resistant *E. faecalis* isolates. However, the *in vitro* antimicrobial activity of Erava in clinical *E. faecalis* isolates from China has not been comprehensive investigated.

In our earlier work, we described Erava heteroresistance in *Staphylococcus aureus* and *Klebsiella pneumonia* isolates with comparatively low Erava minimum inhibitory concentrations (MICs), which suggested that the resistance emerged as a result of antibiotic treatment failure (Zhang F. et al., 2018; Zheng J. X. et al., 2018). However, it is not known whether Erava heteroresistance is also found in *E. faecalis*. Recent evidence has attributed the development of resistance to the new class of Tet antibiotics including tigecycline (Tige) to genetic mutations in the Tet binding site of the 30S ribosomal subunit, which includes 16S rRNA and 30S ribosome protein S10 (Nguyen et al., 2014; Grossman, 2016). Regulators of several efflux pumps or membrane proteins, such as SoxS, MarA, RamA, and Rob, have also been implicated in Tige resistance (Nguyen et al., 2014; Grossman, 2016), although the mechanistic details are not well-understood. In addition, *tet(X)* orthologs, which encode Tet destructases and inactivate Tet antibiotics, represent a unique enzymatic Tet resistance mechanism (Grossman, 2016; He et al., 2019; Sun et al., 2019). Moreover, it is unclear whether Erava susceptibility is influenced by Tet binding site mutations or overexpression of the efflux pump and membrane proteins.

The present study investigated the *in vitro* antimicrobial activity of and potential for the development of heteroresistance to Erava in clinical *E. faecalis* isolates collected at a tertiary care hospital in China. We also examined the influence of Tet resistance genes on Erava susceptibility and analyzed mutations in the 30S ribosomal subunit in Erava heteroresistance-derived clones and *E. faecalis* isolates exhibiting Erava-induced resistance. The genetic and molecular factors affecting Erava susceptibility were assessed by the efflux pump inhibition, qRT-PCR, and *in vitro* overexpression experiments.

## Materials and Methods

### Bacterial Isolates

Non-duplicate clinical isolates of *E. faecalis* (*n* = 276 strains) were collected from January 1, 2011, to December 31, 2015, at the clinical microbiology laboratory of Shenzhen Nanshan People’s Hospital, a tertiary care hospital in Shenzhen, China (Lin et al., 2019). The isolates were from urine, blood, bile, wound exudate, drainage liquid, sputum, and aseptic body fluids and were originally identified and tested for susceptibility to clinically relevant antibiotics using the VITEK 2 Compact automatic microbial analysis system (Biomérieux, Marcy l’Etoile, France). Species-appropriate quality control strains were used to ensure that the isolates met the standards recommended by Clinical and Laboratory Standards Institute (CLSI) guidelines. *E. faecalis* ATCC29212 was used as reference strains for quality control. Procedures involving human participants were performed in accordance with the ethical standards of Shenzhen Nanshan People’s Hospital and the Declaration of Helsinki 1964 and its later amendments. For this particular study, formal consent was not required.

### Antibiotic Susceptibility Testing

Antimicrobial susceptibility to Erava (AdooQ Bioscience, Irvine, CA, United States) was determined by the agar dilution method. MICs of doxycycline, minocycline (Aladdin, Shanghai, China), LZD, and VAN (Macklin, Shanghai, China) were determined by the broth microdilution method according to CLSI guidelines (CLSI-M100-S26). Susceptibility breakpoints of antibiotics, including ampicillin, erythromycin, ciprofloxacin, gentamycin, nitrofurantoin, trimethoprim-sulfamethoxazole, Tet, VAN, and LZD were defined according to CLSI guidelines. The MIC range of Erava for the quality control strain *E. faecalis* ATCC29212 was 0.015–0.06 μg/ml according to CLSI, and the Erava susceptibility breakpoint in *E. faecalis* was defined as ≤0.5 mg/l according to the EUCAST 2019 standard. To characterize antimicrobial activity, MICs of Erava in *E. faecalis* were categorized into four levels (≤0.0625, 0.125, 0.25, and 0.5 mg/l) (Lin et al., 2019).

### PCR Detection of Tet Resistance Genes and 30S Ribosomal Subunit Mutations

Bacterial DNA was extracted using Lysis Buffer for Microorganism to Direct PCR (Takara Bio, Otsu, Japan) according to the manufacturer’s instructions. Tet resistance genes in *E. faecalis* isolates, including *tet(K)*, *tet(L)*, *tet(M)*, *tet(S)*, *tet(O)*, *tet(W)*, *tet(U)*, and *tet(X)* variants, were amplified by PCR, as previously described (Zheng et al., 2017; Bai et al., 2018; He et al., 2019; Lin et al., 2019), using a commercial kit (Takara Bio) according to the manufacturer’s protocol. All primers used are listed in the Supplementary Table S2. Mutations in genes encoding the 30S ribosomal subunit, including four separate copies of the 16S rRNA gene, and the ribosomal protein S10 gene was detected by PCR and sequence alignment using previously described primer sets (Supplementary Table S3) (Lin et al., 2019).

### Population Analysis Profiling (PAP)

PAP experiments were performed as previously described (Zhang F. et al., 2018; Zheng J. X. et al., 2018; Lin et al., 2019), with minor modifications. Briefly, 50 μl aliquots (∼10^8^ CFU/ml) were spread onto Müller-Hinton (MH) agar plates containing various concentrations of Erava (0.25, 0.5, 1.0, and 2.0 mg/l). Colonies were counted after incubation at 37°C for 24 h. Erava heteroresistance was defined as an Erava-susceptible isolate (MIC ≤ 0.5 mg/l) with subpopulations growing in the presence of 0.5 mg/l Erava and with a detection limit of 5 CFU/plate. Three resistant clones were randomly selected from the plates for experiments. The MICs of Erava and Tige were determined as described above (Zhang F. et al., 2018; Zheng J. X. et al., 2018). The frequency of Erava heteroresistance was separately statistically analyzed for clinical isolates with Erava MICs of ≤0.125 and 0.25 mg/l, respectively.

### Efflux Inhibition

The role of the efflux pump in Erava-heteroresistant *E. fecalis* isolates was evaluated using the efflux pump inhibitors (EPIs) Phe-Arg-β-naphthylamide (PAβN; Sigma-Aldrich, St. Louis, MO, United States) and carbonyl cyanide m-chlorophenylhydrazone (CCCP, Sigma-Aldrich). Erava MICs of Erava-resistant subpopulations of heteroresistant isolates were determined by the agar dilution method in the presence and absence of PAβN (50 mg/l) or CCCP (16 mg/l) (Zhang F. et al., 2018). The inhibition was considered significant if the MIC decreased at least fourfold in the presence of EPIs (Zhang F. et al., 2018; Zheng J. X. et al., 2018).

### *In vitro* Induction of Erava Resistance in *E. faecalis*

Three parental *E. faecalis* isolates, including two clinical isolates (FC1 and FC2), and the OG1RF strain were used to generate Erava-resistant *E. faecalis*. The isolates were serially subcultured in MH broth containing increasing concentrations of Erava, with MIC equivalents as the initial concentration (0.06 mg/l) and 2×, 4×, 8×, 16×, and 32× MICs (Zhang F. et al., 2018; Lin et al., 2019). Strains were serially cultured for four passages before exposure to the next concentration. Isolates from passages at each concentration were selected for three generations on MH plates without antibiotics for further experiments, including the detection of mutations at Tet binding sites in the 30S ribosomal subunit, MIC assays, and quantitative reverse transcription (qRT)-PCR analysis.

### qRT-PCR Analysis

Erava is structurally similar to Omadacycline, another member of the Tet drug class. Our previous study showed that heteroresistance to Omadacycline in *E. faecalis* was potentially linked to 11 genes, i.e., RS11300, RS11485, RS10660, RS06880, RS12140, RS02205, RS05865, RS06145, RS09080, RS12590, and RS00630 (Lin et al., 2019). To determine whether these genes are also involved in Erava resistance in *E. faecalis*, we measured their relative transcript levels in clones exhibiting Erava-induced resistance by qRT-PCR. Total bacterial RNA was extracted using the RNeasy Mini kit (Qiagen GmbH, Hilden, Germany), and cDNA was synthesized using the PrimeScript RT reagent kit (Takara Bio). qRT-PCR was performed as previously described (Zheng J. X. et al., 2018). The *recA* gene was used as an internal control. All primers used for RT-PCR are listed in the Supplementary Table S4. Expression levels of target genes in clinical isolates were normalized to those in *E. faecalis* strain OG1RF.

### Gene Overexpression

Recombinant plasmids for overexpression of RS02205, RS12140, RS06145, RS06880, RS11485, and RS00630 were constructed as previously described (Lin et al., 2019) and separately transformed into one to three Erava-susceptible isolates with comparatively low transcript levels of the corresponding genes. All primers used for generating constructs in the present study are listed in the Supplementary Table S5. The MICs and heteroresistance of derivative isolates were evaluated after overexpression of the candidate genes were confirmed by qRT-PCR.

### Statistical Analysis

Continuous variables were analyzed with the Student’s t test and by one-way factorial analysis of variance using SPSS v17.0 software (SPSS Inc., Chicago, IL, United States). Differences were considered statistically significant at *P* < 0.05.

## Results

### *In vitro* Antimicrobial Activity of Erava Against Clinical *E. faecalis* Isolates From China

The distribution of Erava MICs against *E. faecalis* is shown in Table 1, with a focus on isolates resistant to the commonly used antibiotics LZD, VAN, ampicillin, gentamycin, erythromycin, ciprofloxacin, nitrofurantoin, and trimethoprim-sulfamethoxazole. Rates of antimicrobial susceptibility (MIC50/90) and resistance to these antibiotics and Erava are summarized in Supplementary Table S1. The range of Erava MIC values against the 276 clinical *E. faecalis* isolates analyzed in this study was 0.015–0.25 mg/l, and the MIC50/90 was 0.06/0.125 μg/ml. The MIC of Erava was lower than those of doxycycline and minocycline. Notably, Erava MIC was 0.03/0.125 mg/l against multidrug resistant *E. faecalis*, including 51 LZD-intermediate, 13 LZD-resistant, and two VAN-intermediate isolates. In addition, the MIC50/MIC90 of Erava was 0.06/0.06–0.125 mg/l against *E. faecalis* harboring one or more Tet resistance genes, including *tet(M)* and *tet(L)* (Table 2). Erava MIC for *E. faecalis* isolates harboring Tet resistance genes was ≤0.25 mg/l, indicating that Erava can overcome the antimicrobial resistance conferred by Tet resistance genes.

**TABLE 1:** MIC distributions for Eravacycline against *E. faecalis* resistant to commonly used antibiotics.

**Groups (no. of isolates)**	**MIC (mg/l) distribution (N) for Erava**				
	
	**≤0.015**	**0.03**	**0.06**	**0.125**	**0.25**
Total isolates (*n* = 276)	3	53	175	40	5
Linezolid-intermediate *E. faecalis* (*n* = 51)	2	8	34	6	1
Linezolid-resistant *E. faecalis* (*n* = 13)	0	2	9	2	0
Vancomycin-intermediate *E. faecalis* (*n* = 2)	0	1	1	0	0
Ampicillin-resistant *E. faecalis* (*n* = 1)	1	0	0	0	0
Gentamycin-resistant *E. faecalis* (*n* = 159)	1	31	101	24	2
Erythromycin-resistant *E. faecalis* (*n* = 205)	1	42	130	28	4
Ciprofloxacin-resistant *E. faecalis* (*n* = 59)	0	10	37	10	2
Nitrofurantoin-resistant *E. faecalis* (*n* = 2)	0	0	1	1	0
Tigecycline-resistant *E. faecalis* (*n* = 1)	0	0	1	0	0
Trimethoprim-/sulfamethoxazole-resistant *E. faecalis* (*n* = 29)	0	4	18	6	1

*Erava, Eravacycline; MIC, minimum inhibitory concentration.*

**TABLE 2:** Antimicrobial activity of Eravacycline against *E. faecalis* harboring tetracycline resistance genes.

**Tetracycline resistance gene(s)**	**No. of isolates**	**MIC range (mg/l), MIC_50/90_ (mg/l)**					
	
		**Eravacycline**		**Doxycycline**		**Minocycline**	
		**MIC range**	**MIC_50/90_**	**MIC range**	**MIC_50/90_**	**MIC range**	**MIC_50/90_**
*tet(M)*	162	0.015–0.25	0.06/0.125	0.25–32	16/32	0.25–32	16/32
*tet(L)*	5	0.03–0.06	0.06/0.06	0.125–32	16/32	0.125–32	16/32
*tet(M)*, *tet(L)*	60	0.03–0.125	0.06/0.125	4–32	32/32	0.25–32	16/32
*tet(M)*, *tet(K)*	4	0.03–0.06	0.06/0.06	16	16/16	8–16	8/16
*tet(M)*, *tet(L)*, *tet(K)*	1	0.06	0.06	32	32	16	16
-^*a*^	44	0.015–0.25	0.06/0.125	0.125–32	0.5/16	0.125–32	0.25/16

**-^*a*^, E. faecalis* strain negative for the tetracycline resistance genes. MIC, minimum inhibitory concentration.*

### Frequency and Characteristics of Erava Heteroresistance in Clinical *E. faecalis* Isolates

Erava MICs were categorized into three levels – i.e., < 0.125, 0.125, and 0.25 mg/l – and the rate of Erava heteroresistance in *E. faecalis* with various MIC levels was evaluated by PAP. Erava heteroresistance was detected in 0.43% (1/231) and 7.5% (3/40) of isolates with Erava MICs of <0.125 and 0.125 mg/l, respectively. No Erava heteroresistance was observed among isolates with an MIC of 0.25 mg/l. Three heteroresistance-derived clones were selected for further analysis. MIC ranges of Erava and Tige were similarly elevated in these clones (0.5–4 mg/l for Erava and 0.5–8 mg/l for Tige) (Table 3).

**TABLE 3:** MIC values and characteristics of Erava heteroresistance-derived clones.

**Strain**	**MIC (mg/l)**		**MIC for the effect of EPI (mg/l)**				**30S ribosome subunit mutation**
			
	**Erava**	**Tige**	**Erava + CCCP**	**Erava + PAβN**	**Tige + CCCP**	**Tige + PAβN**	
EF16C62-H1	0.5	0.5	0.06	0.25	≤0.015	0.5	−
EF16C62-H2	0.5	1	0.06	0.5	≤0.015	0.5	−
EF16C62-H3	0.5	1	0.06	0.5	≤0.015	0.5	−
EF16C302-H1	2	8	0.25	2	0.25	≥4	−
EF16C302-H2	1	8	0.25	2	0.12	≥4	−
EF16C302-H3	2	8	0.12	2	0.12	≥4	−
EF16C75-H1	2	8	0.12	2	0.12	≥4	−
EF16C75-H2	2	8	0.12	2	0.12	≥4	−
EF16C75-H3	2	8	0.12	2	0.12	≥4	−
EF16C74-H1	0.5	1	0.06	2	≤0.015	1	−
EF16C74-H2	4	8	0.06	2	≤0.015	≥4	−
EF16C74-H3	4	8	0.06	2	≤0.015	≥4	−

*CCCP, carbonyl cyanide m-chlorophenylhydrazone; EPI, efflux pump inhibitor; Erava, Eravacycline; MIC, minimum inhibitory concentration; PaβN, Phe-Arg-β-naphthylamide; Tige, tigecycline.*

To investigate the mechanism of Erava heteroresistance in *E. faecalis*, we screened for mutations in the Tet binding site of the 30S ribosomal subunit. However, there were no mutations in four copies of the 16S rRNA and 30 ribsomal protein S10 genes in heteroresistance-derived clones (Table 3). Moreover, Erava and Tige MICs were four times lower in clones treated with CCCP, although PAβN had no effect (Table 3), indicating that efflux pumps or membrane proteins contribute to the emergence of Erava heteroresistance.

### Mechanism of Erava-Induced Resistance in *E. faecalis*

To clarify the mechanism of Erava resistance in *E. faecalis*, resistance was induced *in vitro* under Erava selection pressure in strain OG1RF and two clinical isolates (FC1 and FC2). The MICs of Erava and Tige as well as mutations in the 30S ribosomal subunit were characterized in the resistant isolates. Erava and Tige MICs showed an increasing trend in *E. faecalis* isolates exhibiting Erava-induced resistance (Table 4); the increase in Erava MICs was accompanied by a higher frequency of mutations in the 16S rRNA gene. The mutation site varied across the four 16S rRNA gene copies, with high frequencies of the A1461G polymorphism in RR2 and of C1265T and C1191T in RR3. In addition, a K57Q amino acid substitution was a mutation hotspot in 30S ribosomal protein S10 of Erava-resistant *E. faecalis*.

**TABLE 4:** MIC values and mutations in tetracycline binding sites in *E. faecalis* isolates exhibiting Erava-induced resistance.

**Strain**	**MIC (mg/l)**		**Mutation in 30S ribosome subunit gene**				
		
	**Tige**	**Era**	RR1*	RR2*	RR3*	RR4*	**S10^#^**
FC1	0.125	0.06	W	W	W	W	W
FC1-1	2	2	W	W	C1265T	W	W
FC1-2	64	16	W	A1461G	C1265T	A1148G	W
					C1191T		
FC1-E32	64	16	W	A1461G	C1265T	W	K57Q
					C1191T		
FC2	0.125	0.06	W	W	W	W	W
FC2-1	2	2	W	A1461G	W	W	W
FC2-2	32	16	W	A1461G	C1265T	W	K57Q
					C1191T		
FC2-E32	32	16	A979T	A1461G	C1265T	G868A	K57Q
			T980A		C1191T	G860A	
OG1RF	0.125	0.06	W	W	W	W	W
OG1RF-E4	32	16	G734C	GGACA	C1265T	A231G	53–56 aa del (RATH)
			G1075T	983–987	C1191T	A1148G	
				CGGAC			
OG1RF-E32	64	16	G734C	C1055G	C1265T	A231G	53–56 aa del (RATH)
			G1075T		C1191T	A1148G	

*Four copies of 16S rRNA; ^#^30S ribosomal protein S10. aa, amino acid; del, deletion; Era, Eravacycline; MIC, minimum inhibitory concentration; Tige, tigecycline.

### Expression Analysis of Candidate Genes Involved in Erava Susceptibility

**FIGURE 1:**
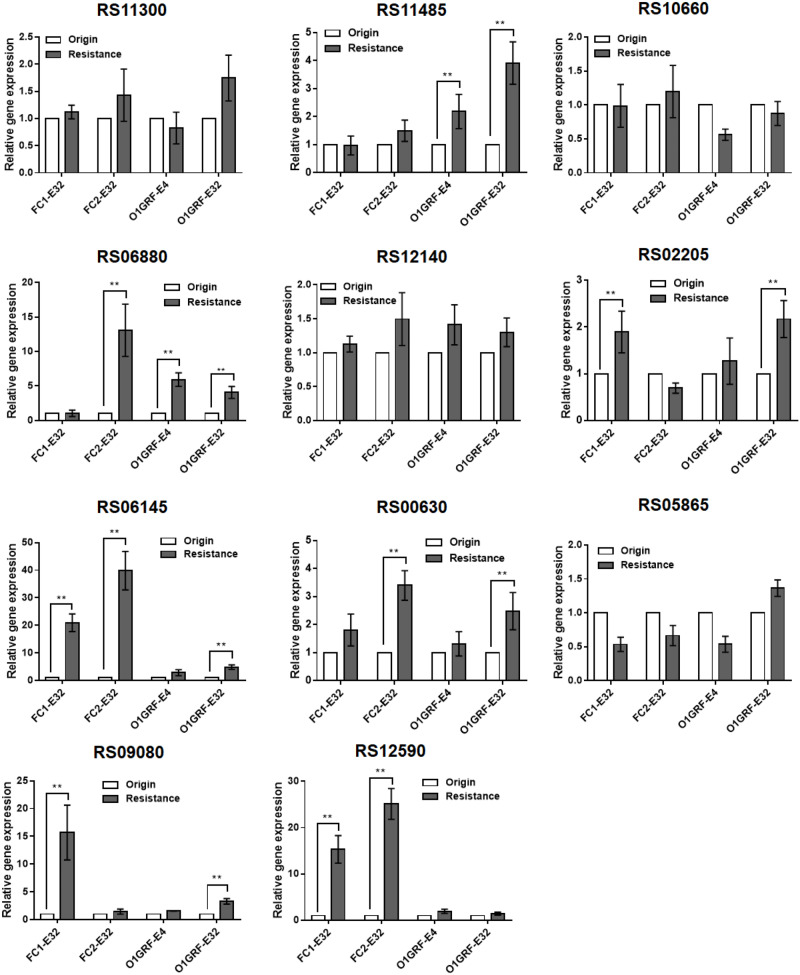
Correlation between transcript levels of 11 candidate genes and Erava-induced resistance in *E. faecalis* isolates. Transcript levels were determined by qRT-PCR, with the housekeeping gene *recA* serving as an internal control. The original (wild-type) strain was used as the reference (expression = 1.0). The *Y* axis represents relative fold change compared to the reference. Resistance, the expression level of corresponding gene in the resistant strain; origin, the expression level of corresponding gene in the origin strain. Resistant isolates and the reference strain are shown in Table 4. Data represent mean ± SD of three technical repeats. **P < 0.01 (Student’s *t*-test).

Compared to the parent strains, the relative expression of six of the candidate genes (RS02205, RS12140, RS06145, RS06880, RS11485, and RS00630) was upregulated in the Erava-heteroresistant derivatives (Figure 1), suggesting that these genes might contribute to Erava resistance in *E. faecalis*.

### Overexpression of Candidate Resistance Genes in *E. faecalis* Confers Erava Heteroresistance

To explore the functional significance of the upregulated candidate genes in Erava heteroresistance, we transformed clinical *E. faecalis* isolates with low endogenous expression of the target genes with recombinant overexpression plasmids and evaluated their susceptibility to Erava and Tige. Target gene overexpression was confirmed by qRT-PCR. Introduction of the exogenous genes did not increase Erava or Tige MIC in the absence of antibiotic (Table 5). However, PAP results showed that overexpression of OG1RF_RS00630 encoding a bone morphogenetic protein (BMP) family ATP-binding cassette (ABC) transporter substrate-binding protein conferred Erava and Tige heteroresistance compared to the parent isolate. In addition, overexpression of OG1RF_RS06145 encoding a molybdate ABC transporter substrate-binding protein, OG1RF_RS12140 encoding an ABC transporter ATP-binding protein, and OG1RF_RS06880 encoding a coenzyme A-binding protein conferred heteroresistance to Tige but not Erava.

**TABLE 5:** Tige and Erava MICs in *E. faecalis* derivatives overexpressing candidate genes and their influence on PAP.

**Transformed plasmid**	**Strain**	**MIC (mg/l)**			
		**Parent isolates**		**Derivative isolates**		**PAP test results for derivative isolates**			
				
		**Tige**	**Erava**	**Tige**	**Erava**	**Tige**	**Erava**
pRS00630	EF16C2	0.125	0.125	0.125	0.125	Positive	Positive
	EF16C284	0.0625	0.125	0.0625	0.125	Positive	Positive
pRS02205	EF16C2	0.125	0.0625	0.125	0.0625	0	0
	EF16C105	0.0625	0.125	0.0625	0.125	0	0
pRS06145	EF16C283	0.0625	0.125	0.0625	0.125	Positive	0
pRS06880	EF16C284	0.0625	0.125	0.0625	0.125	Positive	0
pRS11485	EF16C2	0.125	0.125	0.125	0.125	0	0
	EF16C105	0.0625	0.125	0.0625	0.125	0	0
	EF16C39	0.125	0.125	0.125	0.125	0	0
pRS12140	EF16C105	0.0625	0.125	0.0625	0.125	Positive	0
	EF16C39	0.125	0.125	0.125	0.125	Positive	0

*Erava, Eravacycline; MIC, minimum inhibitory concentration; PAP, population analysis profiling; Tige, tigecycline.*

## Discussion

In this study, we investigated the *in vitro* antimicrobial activity of Erava against 276 non-duplicate clinical *E. faecalis* isolates from China. Consistent with the *in vitro* antimicrobial activity analyses results of Erava against clinical isolates collected globally (Hackel et al., 2013; Sutcliffe et al., 2013; Olesky et al., 2017), in Canada (Zhanel et al., 2018), and the USA (Bouchillon et al., 2018), the overall Erava MIC was ≤0.25 μg/ml against multidrug-resistant *E. faecalis* with intermediate or complete resistance to LZD and intermediate resistance to Vancomycin. Previous studies have seldom reported Tet-resistant *E. faecalis* or *E. faecium* with Erava MIC90 ≤0.125 and 0.06 μg/ml, respectively (Sutcliffe et al., 2013; Bai et al., 2018; Lan et al., 2019; McCarthy, 2019; Morrissey et al., 2019; Wang et al., 2019). Two primary mechanisms known to confer acquired resistance to Tet, the presence of Tet resistance genes encoding efflux pumps *tet(K)* and *tet(L)* and ribosomal protection proteins *tet(M)*, had minimal or no effect on the *in vitro* antibacterial activity of Erava against clinical *E. faecalis* isolates. This likely stems from the fact that Erava have the potential to overcome these mechanisms (Grossman et al., 2012). Our results further confirm that Erava could be effective in the treatment of infectious diseases caused by *E. faecalis*, including isolates harboring one or more Tet resistance genes, including *tet(M)* and *tet(L).* Nevertheless, other Tet resistance genes, such as *tet(U)* and *tet(X)*, involved in two other Mechanism of Tet resistance were not detected in our study and need to be further investigated in *E. faecalis*.

Our previous studies on the rate of Erava heteroresistance in *K. pneumonia* and *S. aureus* provided a basis for evaluating the risk of Erava resistance development (Zhang F. et al., 2018; Zheng J. X. et al., 2018). Our present findings further demonstrate that increasing Erava MICs in clinical *E. faecalis* isolates are associated with enhanced risk of heteroresistance. The concomitant increases in Erava and Tige MICs in clones exhibiting Erava-induced heteroresistance imply that Erava exposure leads to cross resistance to Tige. Thus, although Erava has relatively low MICs against clinical *E. faecalis* isolates, surveillance and early detection of Erava resistance is important; moreover, screening for Erava heteroresistance in clinical *E. faecalis* isolates should be considered as a routine diagnostic procedure.

Mutations affecting Tet binding to the 30S ribosomal subunit is a major factor responsible for both Tet and Tige resistance in several bacterial species (Nguyen et al., 2014; Grossman, 2016). As no mutations were detected in the Tet binding site of the 30S ribosomal subunit in Erava heteroresistant *E. faecalis* in the present work, it is likely that resistance to Tet and Erava involve distinct molecular mechanisms. However, mutations in the 16S rRNA gene and 30S ribosomal protein S10 are frequently detected in *E. faecalis* isolates exhibiting Erava-induced resistance, suggesting that they are contributing factors. Especially the mutation of C1191T in 16S rRNA RR3, which also occurred frequently in Omadacycline-induced resistance strains with MIC ≥ 8 mg/l (Lin et al., 2019), was only present in isolates with Erava MIC of 16 mg/l, suggesting that this site might play a major role in the occurrence of Erava resistance as well as Omadacycline resistance. It is intriguing to examine the impact of direct base mutagenesis on Erava or Omadacycline MIC to confirm the possibility. It has been demonstrated, however, that mutation of *rpsJ* encoding ribosomal protein S10 was sufficient to confer Tet resistance. It is worth noting that the mutation sites of Erava-induced resistant *E. faecalis* isolates were only present in residues 53–57. Of particular interest, a modification (R53Q-Δ54–57ATHK) that occurred in Tige-resistant *E. faecalis* strain selected *in vitro* resulted in a 4-fold increase in Tige resistance (Beabout et al., 2015). Most recently, Dabul et al. (2019) reported that a deletion of amino acids at position 56–59 (HKYK) was present at three Tige-resistant clinical *E. faecalis* isolates. Furthermore, our previous study also proposed that two amino acid substitutions H56Y and K57R appeared in *E. faecalis* OG1RF derivative isolates resistant to Omadacycline, another Tet class of antimicrobial drugs (Lin et al., 2019). Besides, plenty of studies have established that changes or deletions in residues 53–60 in the 30S ribosomal subunit protein S10 were linked to Tet resistance in Gram-positive bacteria *Bacillus subtilis*, *Enterococcus faecium*, and *S. aureus* and in Gram-negative bacteria *E. coli, Acinetobacter baumannii*, *Neisseria gonorrhoeae*, and *K. pneumoniae* (Williams and Smith, 1979; Wei and Bechhofer, 2002; Hu et al., 2005; Villa et al., 2014; Cattoir et al., 2015; Niebel et al., 2015). It has been proposed that this region of the S10 protein near the Tet binding pocket and its mutation may alter the interaction of Tets and 16S rRNA (Brodersen et al., 2000; Hu et al., 2005). Here, mutations, including a K57Q substitution and deletion of 53–56 (RATH), at the same region of the S10 protein were observed in Erava-induced resistant *E. faecalis* isolates, highlighting the relevance of alteration in this specific region of S10 protein for Tets including Erava resistance in *E. faecalis*.

Moreover, the increases in Erava and Tige MICs were reversed in the presence of CCCP (Table 3), indicating that efflux pumps or membrane proteins participate in the early phase of heteroresistance emergence. We previously showed that efflux pumps AcrAB, OqxAB, and MacAB contribute to the Erava resistant phenotype in *K. pneumonia* (Zheng J. X. et al., 2018). The RND-type efflux pump AcrEF was observed overexpression in two Erava-resistant *K. pneumonia* isolates (Zheng J. X. et al., 2018). Besides, adeB is involved in the regulation of efflux pump adeABC, and the Erava MIC in *adeB*-hyperexpressing *A. baumannii* was shown to be reduced by eightfold by disrupting the gene *adeB* (Abdallah et al., 2015). Similar to that of Omadacycline, our current results provide further evidence that ATP-binding cassette family efflux protein RS00630 overexpression contributes to heteroresistance to both Erava and Tige in *E. faecalis*. However, additional studies are needed to clarify the Erava resistance mechanism of RS00630. Although several other efflux pump coding genes were overexpressed in parts of Erava-induced resistance strains, the transgenic overexpression of these genes did not confer Erava resistance or heteroresistance. It turns out that these candidate genes do not correlate to Erava resistance. In addition, the clinical isolates tested in our study were limited by area and country, as these isolates were exclusively collected in one hospital. Therefore, it is important that other isolates are utilized to verify our work to understand the efficacy and underlying the resistance mechanism of these new antibiotics.

## Conclusion

Erava showed excellent *in vitro* antimicrobial effects against clinical isolates of *E. faecalis* in China, including LZD non-susceptible strains with significantly lower MICs than doxycycline or minocycline. However, Erava heteroresistance may occur in *E. faecalis* isolates with MIC ≥ 0.125 mg/l. Overexpression of RS00630 encoding a BMP family ABC transporter substrate-binding protein in *E. faecalis* enhanced heteroresistance to both Erava and Tige, whereas RS12140, RS06145, and RS06880 were associated with heteroresistance to Tige only. Additional studies are needed to determine how RS00630 mediates antibiotic heteroresistance in *E. faecalis*. Our findings suggest that the emergence of Erava heteroresistance in *E. faecalis* clinical isolates – especially those with high Erava MICs – should be closely monitored.

## Data Availability Statement

All datasets generated for this study are included in the article/Supplementary Material.

## Ethics Statement

Procedures involving human participants were performed in accordance with the ethical standards of Shenzhen Nanshan People’s Hospital and the Declaration of Helsinki 1964 and its later amendments. For this particular study, formal consent was not required.

## Author Contributions

ZY and QD conceived and designed the project. ZW, YS, and GX performed the molecular biology experiments and PAP tests. ZP, ZL, and BB performed the efflux inhibition experiment. ZC and JZ performed the MIC test. ZY, QD, and ZW wrote the manuscript with input from all other authors. All authors participated in data analysis.

## Conflict of Interest

The authors declare that the research was conducted in the absence of any commercial or financial relationships that could be construed as a potential conflict of interest.
