# Supplementary material for: Mechanism of Eravacycline Resistance in Clinical Enterococcus faecalis Isolates From China
Source: Front Microbiol. 2020 May 25;11:916. doi: 10.3389/fmicb.2020.00916 (PMC7261854; doi:10.3389/fmicb.2020.00916)
Supplement: Supplementary file 1 [file Data_Sheet_1.PDF]

TABLE S1 | Antimicrobial activity of Erava and comparators against *E. faecalis*

**Organism (no. of isolates)**	**MIC (mg/l)**			**Resistance rate (%)**
	**MIC range**	**MIC_{50}**	**MIC_{90}**	
Total *E. faecalis* (n=276)				
Eravacycline	0.015–0.25	0.06	0.125	−
Doxycycline	0.125–32	16	32	79.3
Minocycline	0.125–32	16	32	73.9
Linezolid	0.25–16	2	4	4.7
Vancomycin	0.25–8	1	2	0
Linezolid-intermediate *E. faecalis* (n=51)				
Eravacycline	0.015–0.25	0.06	0.125	−
Doxycycline	0.125–32	16	32	88.2
Minocycline	0.125–32	16	32	86.3
Vancomycin	0.5–4	1	2	0
Linezolid-resistant *E. faecalis* (n=13)				
Eravacycline	0.03–0.125	0.06	0.125	−
Doxycycline	0.5–32	16	32	84.6
Minocycline	0.125–32	16	16	69.2
Vancomycin	0.25–4	1	2	0
Vancomycin-intermediate *E. faecalis* (n=2)				
Eravacycline	0.03–0.06	0.03	0.06	−
Doxycycline	0.25–16	0.25	16	50
Minocycline	0.5–16	0.5	16	50
Linezolid	2	2	2	0

−, Not counted due to a lack of a clear cutoff value.
Erava, Eravacycline; MIC, minimum inhibitory concentration.

TABLE S2 | Primers used for Tet resistance genes amplification

**Target gene**	**Primer name**	**Primer sequence (5'-3')**	**Amplicon size (bp)**	**Reference**
tet(M)	*tet(M)*-F	CAATACAATAGGAGCAAGC	974	Lin et al., 2019
	tet(M)-R	CGAACAAGAGGAAAGCATAAG		
tet(L)	*tet(L)*-F	GTAACCAGCCAACTAATGAC	908	Lin et al., 2019
	tet(L)-R	TTGGATCGATAGTAGCC		
tet(K)	*tet(K)*-F	TCGATAGGAACAGCAGTA	169	Collins et al., 2016
	tet(K)-R	CAGCAGATCCTACTCCTT		
tet(S)	*tet(S)*-F	TACCTCCATTTGGACCTCAC	1050	Lin et al., 2019
	tet(S)-R	GAACGCCAGAGAGGTATT		
tet(W)	*tet(W)*-F	GAGAGCCTGCTATATGCCAGC	168	Collins et al., 2016
	tet(W)-R	GGGCGTATCCACAATGTTAAC		
tet(O)	*tet(O)*-F	AACTTAGGCATTCTGGCTCAC	515	Collins et al., 2016
	tet(O)-R	TCCCACTGTTCCATATCGTCA		
tet(U)	*tet(U)*-F	CAAAAGAAATCGATACGTGG	294	Nishimoto et al., 2005
	tet(U)-R	CGTCTGCAGATTCCTTAAAAGTC		
tet(X) and tet(X2)	*tet(X)*-F	GGAAACCGGCTAATGGCAT	230	He et al., 2019
	tet(X)-R	AATCCTACAAATGACAACGTCG		
tet(X3)	*tet(X3)*-F	TAATGGCGGGACATCAGG	294	He et al., 2019
	tet(X3)-R	AGGCGACATCAAATGAGCAG		
tet(X4)	*tet(X4)*-F	CCGATATTCATCATCCAGAGG	328	He et al., 2019
	tet(X4)-R	CGCTTACTTTTCCAAGACTTACCT		

TABLE S3 | Primers used for amplification of 30S ribosomal subunits of *E. faecalis*

**Target gene**	**Primer name**	**Primer sequence (5'-3')**	**Amplicon size (bp)**	**Reference**
RR1	RR1-F	CCGGAGGATTAGGTATTGGGATTGA	2356	Lin et al., 2019
	RR1-R	CTCAAAACTGAACAAAGTAAAGACG		
RR2	RR2-F	CTGAACAAGTGGACACCCAA	2767	Lin et al., 2019
	RR2-R	CTCAAAACTGAACAAAGTAAAGACG		
RR3	RR3-F	CGAATAGAAATGCTTGGGAAC	2995	Lin et al., 2019
	RR3-R	CTCAAAACTGAACAAAGTAAAGACG		
RR4	RR4-F	GTAGCCCAAGAAGAAACC	2668	Lin et al., 2019
	RR4-R	CTCAAAACTGAACAAAGTAAAGACG		
S10	S10-F	GACACGCTCGGACGCTTTGCCATGA	1708	Lin et al., 2019
	S10-R	AATTCCATTATGCAAGCAACCTCCTC		

RR1-RR4: four copies of 16S rRNA gene; S10: 30S ribosomal subunit protein S10 in *E. faecalis*.

TABLE S4 | Primers for qRT-PCR in this study

Target gene	Primer	Primer sequence (5'-3')	Amplicon size (bp)
recA	recA-F	CGACTAATGTCTCAAGCACTAC	106
	recA-R	CGAACATCACGCCAACTT	
RS12140	RS12140-F	ACACTAATCCACAACACAT	112
	RS12140-R	TACTCTTGCTCAACTCTTC	
RS02205	RS02205-F	TCTCAATGAACAAGCGGTAA	102
	RS02205-R	GCCACGGTTAAGTTAGGAA	
RS06145	RS06145-F	GCATCCAGATATTCAGGTA	195
	RS06145-R	TTAGGCACAATAAGAACAAG	
RS11485	RS11485-F	TTGCCTTAGTGTTAATCAT	107
	RS11485-R	AACGGCTAATAGAAGAAC	
RS06880	RS06880-F	GCATTAACAGAGATTCCT	179
	RS06880-R	ATAATAACTTGACGACCTT	
RS00630	RS00630-F	GTATTGGCTACTTGCTAA	136
	RS00630-R	GCTGCTTCATTATCTCTAA	
RS11300	RS11300-F	CTTGTGGTAACGGTAATG	113
	RS11300-R	TAACGCTTCTTCTTGAAC	
RS10660	RS10660-F	ACGAGCCAACAAATCATT	121
	RS10660-R	TCAACCACTTCATCAACAA	
RS05865	RS05865-F	ATTACCGACAGGCAACTT	134
	RS05865-R	TTCCAGGTGCTAATAGATTCA	
RS09080	RS09080-F	AGCAAGTTACGAGATGTA	200
	RS09080-R	GTTGATAGGCACCATATAC	
RS12590	RS12590-F	GGCATCAACCTTGGCTGTA	95
	RS12590-R	GACTGGAATCAACGCAATCAC	

TABLE S5 | Primers used for construction of gene overexpression strains

Target gene	Primer	Sequence (5'-3')	Product length (bp)	Underline (enzyme site)
**Construction of the overexpression plasmids**				
RS00630	RS00630-F	CGCGGATCCCTATTTTTAGGAGGCTTTACAG	1152	BamHI
	RS00630-R	CCGCTCGAGGGAACAATAATGAGCCCTTCT		XhoI
RS02205	RS02205-F	TCCCCCGGGAAGAAGTGAAAGATGCGGTGA	1003	SmaI
	RS02205-R	CCGCTCGAGAAGTTTCAATCAGTGCTTGCA		XhoI
RS06145	RS06145-F	CGCGGATCCTGACTTGAGTTATGGCGGGTA	867	BamHI
	RS06145-R	CCGCTCGAGAAGAATTGGTCGAACATCCAT		XhoI
RS06880	RS06880-F	CGCGGATCCCCAAAACCCTTTACTCAGCGT	529	BamHI
	RS06880-R	CCGCTCGAGAGCTTTCCGTTCAGGACGCTC		XhoI
RS11485	RS11485-F	CGCGGATCCGTTGTCTTTTAAACGAGAGAG	962	BamHI
	RS11485-R	CCGCTCGAGAAAGTATTCAACGCCCTGAAT		XhoI
RS12140	RS12140-F	CGCGGATCCCATGAAGTTGCACCGAATCAT	1092	BamHI
	RS12140-R	CCGCTCGAGATCACTTGCGGAATCATCAAT		XhoI
**Verification of the overexpression plasmids**				
IDRS00630	IDRS00630-F	TTGACCGTGATCAAGATGCTG	624	
	IDRS00630-R	AGACTGTAACATTCTCACGCA		
IDRS02205	IDRS02205-F	AGTCGATACGCCAGAAGCCAT	623	
	IDRS02205-R	GACTGTAACATTCTCACGCAT		
IDRS06145	IDRS06145-F	AGCGAATGCAAGTGCAGAAGC	559	
	IDRS06145-R	AGACTGTAACATTCTCACGCAT		
IDRS06880	IDRS06880-F	TGCTTGGCGAAACGGTTTATG	604	
	IDRS06880-R	ACTATTGCCGGGATAGACTGT		
IDRS11485	IDRS11485-F	AGCAATAGGCATGGTAATTGT	524	
	IDRS11485-R	ATGCGCCATGACAGCCATGAT		
IDRS12140	IDRS12140-F	ATGTGTGACAACATTGCCATT	628	
	IDRS12140-R	GACTGTAACATTCTCACGCAT		

References

Collins, J. R., Arredondo, A., Roa, A., Valdez, Y., León, R., & Blanc, V. (2016). Periodontal pathogens and tetracycline resistance genes in subgingival biofilm of periodontally healthy and diseased Dominican adults. Clinical oral investigations, 20(2), 349–356. https://doi.org/10.1007/s00784-015-1516-2

He, T., Wang, R., Liu, D., Walsh, T. R., Zhang, R., Lv, Y., Wang, Y., et al. (2019). Emergence of plasmid-mediated high-level tigecycline resistance genes in animals and humans. Nat Microbiol, 4(9), 1450-1456. doi:10.1038/s41564-019-0445-2

Lin, Z., Pu, Z., Xu, G., Bai, B., Chen, Z., Sun, X., Zheng, J., Li, P., Qu, D., Deng, Q., & Yu, Z. (2020). Omadacycline Efficacy against Enterococcus faecalis Isolated in China: In Vitro Activity, Heteroresistance, and Resistance Mechanisms. Antimicrobial agents and chemotherapy, 64(3), e02097-19. https://doi.org/10.1128/AAC.02097-19

Nishimoto, Y., Kobayashi, N., Alam, M. M., Ishino, M., Uehara, N., & Watanabe, N. (2005). Analysis of the prevalence of tetracycline resistance genes in clinical isolates of Enterococcus faecalis and Enterococcus faecium in a Japanese hospital. Microb Drug Resist, 11(2), 146-153. doi:10.1089/mdr.2005.11.146
